# News sharing on Twitter reveals emergent fragmentation of media agenda and persistent polarization

**DOI:** 10.1140/epjds/s13688-022-00360-8

**Published:** 2022-08-19

**Authors:** Tomas Cicchini, Sofia Morena del Pozo, Enzo Tagliazucchi, Pablo Balenzuela

**Affiliations:** 1 Departamento de Física, Facultad de Ciencias Exactas y Naturales, Universidad de Buenos Aires, Av. Cantilo s/n, C1428EGA Ciudad Autónoma de Buenos Aires, Argentina; 2 Instituto del Cálculo (IC), UBA-CONICET, Intendente Güiraldes 2160, C1428EGA Ciudad Autónoma de Buenos Aires, Argentina; 3 Instituto de Física de Buenos Aires (IFIBA), CONICET, Av. Cantilo s/n, C1428EGA Ciudad Autónoma de Buenos Aires, Argentina; 4 Latin American Brain Health Institute (BrainLat), Universidad Adolfo Ibañez, Diagonal Las Torres 2640, 7910000 Santiago, Chile

**Keywords:** Complex networks, Social media, News consumption, Natural language processing

## Abstract

News sharing on social networks reveals how information disseminates among users. This process, constrained by user preferences and social ties, plays a key role in the formation of public opinion. In this work, we used bipartite news-user networks to study the news sharing behavior of main Argentinian media outlets in Twitter. Our objective was to understand the role of political polarization in the emergence of high affinity groups with respect to news sharing. We compared results between years with and without presidential elections, and between groups of politically active and inactive users, the latter serving as a control group. The behavior of users resulted in well-differentiated communities of news articles identified by a unique distribution of media outlets. In particular, the structure of these communities revealed the dominant ideological polarization in Argentina. We also found that users formed two groups identified by their consumption of media outlets, which also displayed a bias towards the two main parties that dominate the political life in Argentina. Overall, our results consistently identified ideological polarization as a main driving force underlying Argentinian news sharing behavior in Twitter.

**Supplementary Information:**

The online version contains supplementary material available at 10.1140/epjds/s13688-022-00360-8.

## Introduction

Mass media plays a preponderant role in the formation of public opinion [1, 2], as an increasing proportion of the population is exposed to media outlets and actively engages in sharing news on social media platforms [3–5]. Therefore, the process of opinion formation is influenced by how information spreads on social media [6, 7]. In turn, this circulation depends on the connectivity between users, reflecting ties based on intellectual or ideological affinity, membership to certain social groups, or shared trust in influential individuals or organizations, among other factors [8–10]. Thus, as these social ties translate into the user connectivity backbone, social networks become clustered and modular, resulting in topological constraints on information flow that can be analyzed using methods from complex network theory.

The emergence of highly connected groups of individuals is a topological feature that repeatedly arises in studies of social networks. This feature has been observed in networks defined by preferential message propagation (*retweet networks*, in the case of Twitter) [11, 12], as well as in networks of followers [13]. High within-group connectivity is usually a reflection of similar user behavior [13–18], leading to homogeneous communities that are known as *echo chambers* [19–21]. The emergence of polarized behavior in social media is frequently related to homogeneous groups of users who extreme their opinions on discussions around specific topics (gun control, vaccination, etc.). However, these topics are rarely discussed in isolation [22] and the phenomenon of issue alignment phenomena plays [23] a key role in the process of polarization, leading to the self-identification of users with antagonistic ideologies.

The role of social media and news sharing in the formation of ideological echo chambers has become an important topic of research in recent years. For instance, in [24] the authors analyzed if exposure to partisan information among Twitter users contributes to the formation of ideological echo chambers. Authors in [25] conducted research to determine whether social media, such as Facebook, are capable of shaping the exposure to ideologically diverse news. Finally, the investigation reported in [26] addressed the relationship between cognitive congruence (i.e., ideology) and news sharing behavior in Brazil, Argentina and USA.

In this paper, we investigated the key features leading to the emergence of well-defined groups in terms of the sharing of news published by Argentinian major media outlets. Our main hypothesis is that individual news sharing behavior on Twitter is driven by personal preferences and ideological affinity, in such a way that it is possible to detect emerging polarized groups as a consequence of social media interactions. Under this hypothesis, we sought to provide a quantitative answer to the following questions: Is news sharing constrained by features related to users or news? Or does this information diffuse freely on social media?. Why are certain groups of news more frequently co-shared among them than with others?. Do users tend to form clusters according to their preferences in news consumption?. Can the news consumption profiles of users be used to define and identify echo-chambers?. Does media consumption in social media reflect the political polarization in Argentina? Does this depend on whether users explicitly share their political preferences, or on whether data is collected during an electoral campaign?

## Background

In this section we sketch the political and media landscape in Argentina during the period under study, with the purpose of contextualizing our analyses and results.

National elections in Argentina have two mandatory instances: the primary election, called PASO (in Spanish: *Primarias, Abiertas, Simultáneas y Obligatorias*; in English: Open, Simultaneous and Obligatory Primaries), and a general election. In 2019 these instances took place on August 11th and October 27th, respectively. If needed, depending on the results of the general elections, a third instance (called *ballotage*) can also take place.

In the last ten years, the political landscape in Argentina was dominated by two coalitions: a center-left coalition led by Cristina Fernandez de Kirchner (called *Frente de Todos*) and a center-right coalition led by Mauricio Macri (called *Juntos por el Cambio*). Cristina Kirchner was the Argentinian president in the periods $2007-2011$ and $2011-2015$ and Mauricio Macri in $2015-2019$ [27].

In the 2019 elections, the candidates of the center-left coalition were *Alberto Fernandez* and *Cristina Fernandez de Kirchner*, while *Mauricio Macri* competed for his re-election as president for the center-right coalition with *Miguel Angel Pichetto* as candidate for vice-president. Also *Axel Kicilliof* and *Maria Eugenia Vidal* were candidates to Governor of the Buenos Aires province for the *Frente de Todos* and the *Juntos por el Cambio* coalitions, respectively.

Concerning the media landscape, the ranking of the most consumed digital media outlets in Argentina is dominated by three players: *Infobae*, *Clarin* and *La Nacion*, with approximately 20 millions of unique users each as of 2020 [28], according to Comscore data. These are followed by a second group of outlets with audiences that oscillate between 6 millions and 13 millions of unique visitors. Among them, we highlight *Pagina 12*, *Ambito Financiero*, *TN Noticias* and *El Destape Web*.

Ideological bias has been identified for some of these media outlets. For instance, *Pagina 12* is considered a left-of-center broadsheet newspaper, *Clarin* is a centrist tabloid, and *La Nacion* is a center-right broadsheet [29]. Also, in [30] authors refer to *La Nacion* as a newspaper which has historically adopted an elitist stance marked by support of the upper classes and their interests, and to *Clarin* as a newspaper with centrist ideology, known for its general perspective and its broad, middle-class target audience.

During the period 2008-2014 there was a confrontation between the government of *Cristina Fernandez de Kirchner* and mass media corporations [30]. This conflict resulted in the emergence of a group of newspapers closer to the policies of the *Kirchner* government (e.g., *Pagina 12*), and in the consolidation of another group of newspapers whose editorials were strongly critical of the measures taken by the administration at this time (e.g., *Clarin* and *La Nacion*, among others) [30–32].

## Data and methods

Twitter users sharing links to media content were selected in order to analyze the emergence of structures from their interactions, following a similar approach than in [3].

### Data

Twitter data was acquired using the official API [33], together with custom developed Python codes. The acquisition process consisted of the following steps:

*User selection: live download*. Twitter activity was downloaded using the Stream Listener tool in the Twitter API. Politically active users were filtered by keywords (discarding retweets) associated with politicians, electoral alliances and political parties (see keywords in see Additional file 1) during the primary 2019 Argentinian elections (between August 5th and August 12th). Also, a control group was selected using keywords associated to the name of the main media outlets in Argentina (also discarding retweets) during the period from August 29th to September 30th 2019 and from June 4th to July 4th 2020. We collected a similar number of users for both datasets: 38*K* for 2019 and 35*K* for 2020.

*Twitter activity: tweet download*. Full twitter activity corresponding to both datasets was downloaded during two different periods of time: from August 29th to September 30th 2019 and from June 4th to July 4th 2020. We collected 1,368,914 tweets of politically active users in 2019 and 987,271 tweets in 2020. The control dataset comprised 511,308 tweets in 2019 and 576,137 tweets in 2020.

*Embedded* URLs *in tweets*: From previously downloaded tweets, we keep only those containing embedded *URLs*.

*URL filter*: Outlet domains were obtained from the *URLs* together with an Argentinian media database provided by ABYZ News Links [34]. We only keep tweets from the major twenty Argentinian media outlets, obtaining 80,811 and 66,688 tweets for the dataset comprising politically active users, and 31,811 and 41,593 tweets for the control dataset in 2019 and 2020 respectively.

The bipartite network data is shown in Table 1.

**Table 1:** Bipartite networks description of politically active users and control group datasets

Dataset	Period	# Tweets	# Urls	# Users
Politically active users	2019	80,810	40,188	10,748
	2020	66,687	34,580	10,115
Control group	2019	31,810	14,604	5158
	2020	41,437	17,285	6756

### Methods

#### Bipartite networks and their projections

The complex patterns of news shared by multiple users can be mapped onto bipartite networks following the procedure sketched in [3].

Bipartite networks have two different classes of nodes; in this case, the networks can be projected into news and user layers. Connections in the news projection indicate co-consumption across users, while the user projection describes users connected by news shared by both of them.

Projections of bipartite networks can be implemented in several ways, as for instance following the method developed in [35]. Understanding that projections introduce several noisy edges between nodes of the same layer, such a method proposes a null model to define which edge plays a significant role on the monopartite network structure. Based on this idea, here we follow a similar approach, combining a simple projection method [36] with a significance filter introduced in [37]. As in [3], a hyperbolic projection was used in order to mitigate the influence of highly connected nodes of both layers (see Additional file 1 2). The significance filter maintains those links whose weights are meaningful in comparison to those expected from a null stochastic model that preservers node degree and total strength. The same approach was followed in [38].

Given that projections do not necessarily produce fully connected networks, we kept the largest connected components for further analysis.

#### Networks metrics

We mainly focused on the analysis of collective structures and the role of nodes within these structures.

To detect communities in both projections of the bipartite networks we used a Python implementation of the Louvain algorithm [39, 40] based on the optimization of the modularity *Q*. Due to the stochastic nature of the Louvain algorithm, the obtained community partitions may differ from each other in a comparatively small number of nodes. To obtain a well-defined membership metric of the nodes, we constructed consensus networks [41] which allow the robust assignment of nodes to communities.

In spite of known limitations of community detection based on modularity for the case of weighted networks [42], the obtained results were robust when compared to other algorithms such as Infomap and Label Propagation, as can be seen in Additional file 1 4, showing the normalized mutual information between partitions obtained with different methods.

We also analyzed the role of users in the network by means of the participation coefficient and the within-module degree, as defined in [43]. Given a partition in communities, the within-module degree measures how connected is a node within its own community, while the participation coefficient determines how well connected is a node with respect to the other communities of the network.

#### Nodes metrics

To analyze the properties of emergent structures on both projections of the bipartite networks, we focused on metrics related to semantic content and media outlet membership (for the news projection) and to the user profile of media consumption (for the user projection).

##### News projection

When nodes are identified with media content, two features are relevant for our analysis: the media outlet in which the article was published and its semantic content:

##### Media outlet

We first classify each news article according to the media outlet where it has been published.

##### Semantic content analysis

The following steps were applied to the text of each news article:

- **tokenization**: each element of the corpus was separated in individual terms, and non-alphanumeric characters and punctuation was removed. All terms were converted to lowercase.
- **stopwords filtering**: using the Spanish stopwords database provided by nltk [44], the most common (and thus the least informative) words were filtered out.
- **term basis construction**: a term basis was generated from the set of used terms.
- **frequency description**: each news article was described by a term frequency vector with entries corresponding to the basis computed in the previous step, i.e., the i-th term corresponded to the number of times the i-th term of the basis appeared in the corpus.
- **tf-idf description**: to mitigate bias due to excessive contribution of frequently used words and increase the contribution of unusual (but informative) words, the term frequency - inverse document frequency (tf-idf) statistic was computed [45], resulting in the following value for the i-th element of the vector news representation:
  1 $$ v_{i} = f_{i} \cdot \log \biggl(\frac{N}{N_{i}} \biggr), $$
  where *N* is the number of documents in the corpus and $N_{i}$ is the number of documents where the i-th term appears.

After these processing steps, the news corpus was described as a matrix $M \in \mathrm{R}^{n \times m}$, with *n* the number of documents in the corpus and *m* the number of basis terms.

##### Unsupervised topic detection

Starting from this mathematical representation of the corpus, it is possible to detect the main topics (i.e. groups of similar articles with roughly the same semantic content) by performing Non-negative Matrix Factorization (NMF) [46, 47] on the document-term matrix (M). NMF results in the factorization of the news-terms matrix *M* as the product between two matrices:

2 $$ M \approx N \cdot W , \quad \textit{con} N \in \mathrm{R}^{n \times t} y W \in \mathrm{R}^{t \times m}, $$

where *t* is the chosen number of topics, and *N* and *W* are the resulting matrix dimensions. Both matrices are composed only of positive entries, allowing their straightforward interpretation.

Following the procedure sketched in [48], we define the media agenda as the fraction of articles belonging to each topic.

##### Sentiment analysis

Sentiment analysis was performed in the phrases of each news article where candidates of the main political coalitions appeared mentioned. For this, we used the algorithm developed in [49]. This algorithm assigns a label (+1, 0, −1) depending on whether the sentiment of the sentences is considered positive, neutral or negative, respectively. Using this information, each news article can be characterized by the proportion of phrases with negative, neutral and positive phrases towards each of the candidates. Then, we applied a metric known as Sentiment Bias Statistic (*SB*, defined in [50]), which is computed as follows.

- In each text, we detected phrases mentioning the names of the candidates of the two main coalition disputing the national elections in Argentina 2019 (“Cristina”, “Kirchner”, “Alberto”, “Fernandez” from the center-left, and “Mauricio”, “Macri” or “Pichetto” from the center right).
- We applied sentiment analysis to these sentences and counted the amount of positive, negative, and neutral mentions for each of the candidates.
- For each news article, $\#KF_{+}$ ($\#KF_{-}$) stands for fraction of positive (negative) mentions of *Cristina Kirchner* or *Alberto Fernandez* and $\#MP_{+}$ ($\#MP_{+}$) for positive (negative) mentions of *Mauricio Macri* or *Miguel Angel Pichetto* and the *SB* is defined according to equation (3).

3 $$ SB = (\#KF_{+} - \#KF_{-}) - (\#MP_{+} - \#MP_{-}) . $$

The statistic *SB* is a measure of the bias towards one of the coalitions. If $SB>0$, the bias is positive towards the candidates of the center-left (*Cristina Kirchner* or *Alberto Fernandez*) compared to the candidates of the center-right coalition (*Mauricio Macri* or *Miguel Angel Pichetto*). If $SB<0$ it is the same situation, but towards the center-right coalition.

##### User projection

In this projection, users are linked by the news they jointly consume. We quantified the diversity in the media outlets consumed by each user in terms of the following vector:

4 $$ m^{i} = (1,\ldots,j,\ldots,0), $$

where the $m^{i}_{j}$ component indicates the number of news from the *j* media outlet shared by the *i* user. Given the heterogeneity in the distribution of news outlets in the corpus, we introduced a corrected version of this metric :^1^

5 $$ \bigl(m^{i}_{c}\bigr)_{j} = m^{i}_{j} \cdot \log \biggl(\frac{N}{N_{j}} \biggr), $$

where *N* is the total number of users and $N_{j}$ the total number of users sharing news belonging to the *j* media outlet. Here, the factor $\log (\frac{N}{N_{j}} )$ corrects the potential bias caused by a given article being shared by multiple users.

Given that users can share news from multiple outlets or, conversely, share news from only one of them, we estimated how diverse was the user behavior in terms of the shared media outlets using the maximum value of the consumed media vector. This measures the Lack of Diversity (LD) in the user behavior:

6 $$ \mathrm{LD}_{i} = \max_{j \in M}\bigl\{ \bigl(m^{i}_{c}\bigr)_{j}\bigr\} , $$

where *M* is the total number of media outlets in our data set. After normalization, this lack of diversity lies between 0 and 1.

## Results

Our results aim to provide insight about the underlying mechanism in the formation of tight communities of news articles and users. Our hypothesis is that individual preferences and ideological positioning give rise to communities identified either by the shared media outlets or by the semantic content of the shared news.

First, we briefly summarize the datasets of politically active users in 2019 and 2020. Table 2 contains the final number of nodes for each period. The same data for the control group can be found in Additional file 1 3, together with a full topological characterization of the networks.

**Table 2:** Number of nodes in the users and news networks of the politically active users dataset

Period	Users	News articles
29/07 to 29/08 2019	3625	12,221
4/06 to 4/07 2020	4323	10,975

**Figure 1:**
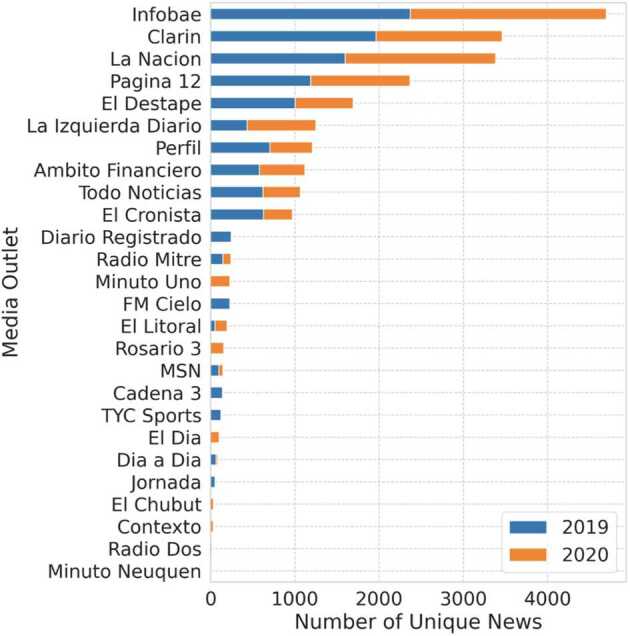
Distribution of news articles according to media outlet. Here we plot the amount of articles of each media outlet corresponding to both analyzed years in the set of politically active users

In Fig. 1 we show the distribution of news articles classified by media outlets for 2019 and 2020. We can see that the distribution of the scrapped news is similar to the level of consumption of the main media outlets [28].

In what follows, we analyze networks emerging from both projections of the bipartite users-news networks.

### The news projection

In this projection, nodes represent news articles and edges are proportional to the number of users co-sharing the corresponding news. We performed a topological analysis of these networks, together with the semantic analysis of news content.

**Figure 2:**
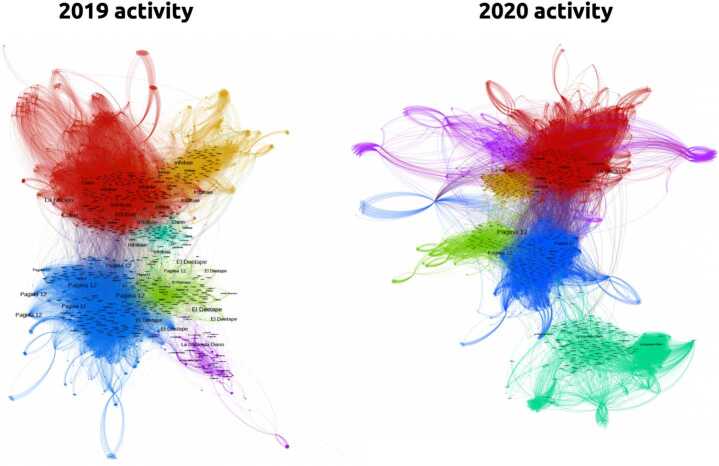
2019 and 2020 news networks visualization. Nodes were colored by community membership and labeled by media outlet proportionally to their within-module degree

We performed community detection in both data sets, yielding the structures visualized in Fig. 2. The displayed labels correspond to the media outlets of the news with the largest within-module degree. Colors represent the different communities. It is possible to appreciate the strong relationship between the news media outlets, the emerging community structure and how it persists across both datasets: *Pagina 12* and *El Destape Web* on one side, *La Nacion*, *Clarin* and *Infobae* on the other.

**Figure 3:**
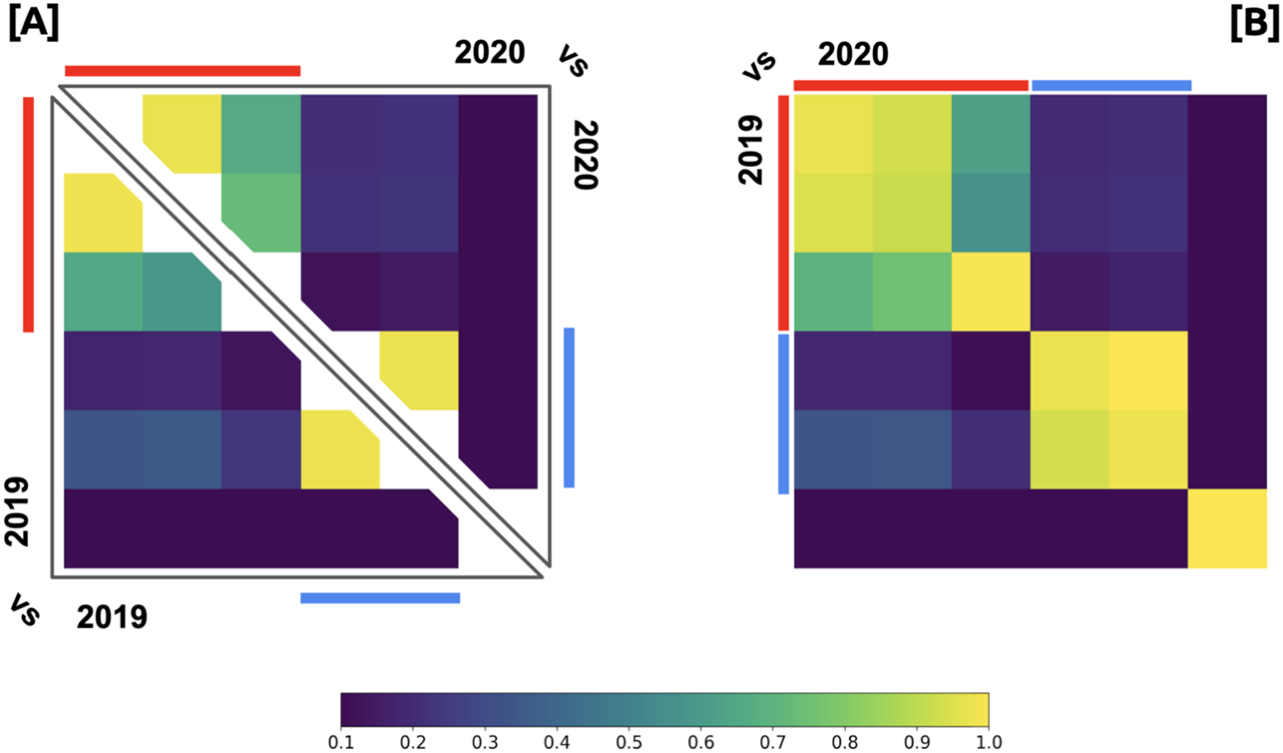
Consistency of news network. Panel [**A**] accounts for similarities among communities of same years and Panel [**B**] compares 2019 against 2020

To quantify the role of media outlets in the emergence of this structure, we described each community by an array $C^{m}_{i}$ where each component accounts for the number of news of media *i* in community *c*. Then, we calculated the cosine similarities between these vectors. The results are shown in Fig. 3. Panel [A] accounts for similarities among communities of the same year and Panel [B] compares 2019 against 2020. Three groups consistently arise in the comparison between both years: a first one composed by three communities, the second one by two communities, and a small group in third place.

The media outlet vector distributions are indicative of a strong polarization between the two main groups of news: the first one is composed by news from mainly three outlets: *Clarín*, *La Nación* and *Infobae*, while the second one is composed mostly by news from *Página 12* and *El Destape*. The third and smallest group is a community with news from *La Izquierda Diario* (the newspaper of the socialist worker party).

**Figure 4:**
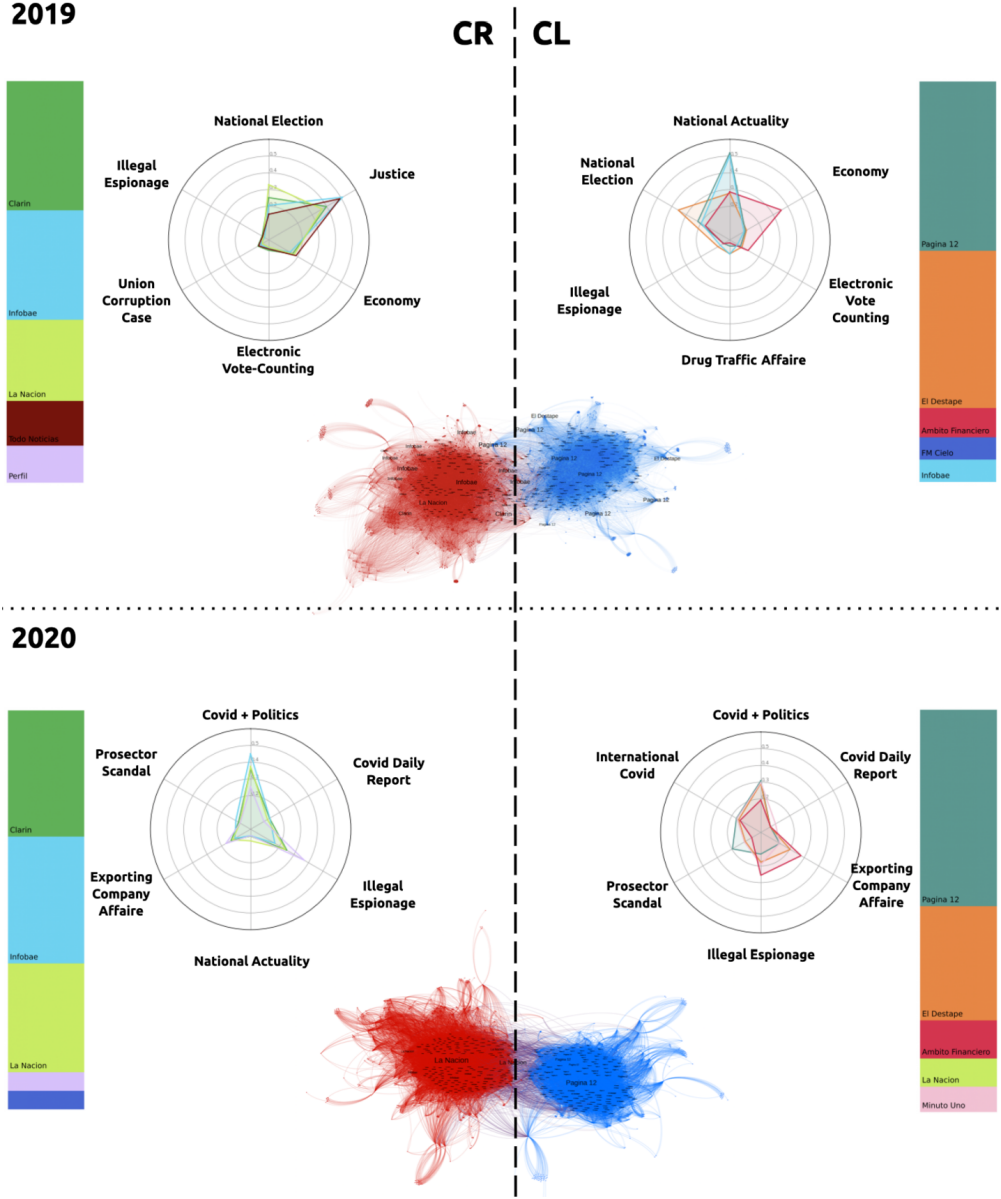
Media outlets distributions and topic decomposition for the 2019 and 2020 two main communities. The stacked bars represent the media outlet distribution, while the radar plots display the media agenda. The agenda of each outlet is indicated with lines colored with the same color as in the stacked bar

We complemented this analysis by performing topic decomposition of the news content within each community (see Methods). Results are detailed in Table 3 for the six main communities of each dataset, with a more detailed version in Fig. 4 for the two largest communities.

**Table 3:** Main communities description for 2019 and 2020 news networks

Community Alias	Nodes (%)	Main Media Outlets	Main Topics
2019			
Center-Right I	18.68	Clarin, Infobae, La Nacion	Justice, National Elections, Economy
Center-Left I	14.75	Pagina 12, El Destape	National Actuality, Economy, National Elections
Center-Left II	8.34	El Destape, Pagina 12	National Actuality, National Election
International	5.5	Infobae	International Actuality
Center-Right II	4.4	Clarin, Infobae, La Nacion, Todo Noticias	Justice, National Election
Radical Left	3.4	Izquierda Diario	Politics, Public Health, Economy
2020			
Center-Right I	16.07	Clarin, Infobae, La Nacion	Covid + Politics, Illegal Espionage
Center-Left I	11.33	El Destape, Pagina 12	Covid + Politics, Exporting Company Affaire, Illegal Espionage
Center-Left II	8.61	Pagina 12, El Destape	National Actuality, Economy, Covid Daily Report
Center-Right II	7.43	Infobae, La Nacion, Clarin	International Covid, Covid Daily Report, Justice
Radical Left	7.1	La Izquierda Diario	llegal Espionage, Globally Known Racial Issue
International	4.5	Infobae	International Actuality

In Fig. 4, we focus the analysis on the two main communities of both years. Here, media outlet distributions are shown in the stacked bars and the media agendas in the topic space depicted in the radar plots. These results highlight that the media outlet distribution remains the same in time, while the media content is dynamic and provides information of what is happening at each specific time point.

Consistent with the media landscape described in the Background section, the media outlets are distributed in two main groups paralleling the two most voted and widespread political coalitions in the 2019 national Argentinian elections. While *Página 12* and *El Destape* are known to support the center-left political party, *Clarín* and *La Nación* are known for supporting the opposing coalition [30–32].

In light of these results, we defined a metric accounting for the support given to the candidates by each media outlet. For this, we first noted that in the topic **National Elections**, present in the two main communities of 2019 dataset, the names of the main candidates are highlighted (see word clouds for each community in Additional file 1): *Cristina*, *Alberto* and *Fernandez* from the Center-Left coalition and *Vidal* and *Macri* from the Center-Right one. Thus, we applied sentiment analysis to phrases mentioning the candidates in media content to measure positive or negative bias towards them, as described in the Methods section.

We calculated the Sentiment Bias (SB) for all media content in the two main groups of communities for 2019 and 2020. The center-left community, dominated by *Página 12* and *El Destape*, gives $SB = 0.17 \pm 0.05 $ in 2019 and $SB =0.02 \pm 0.05$ in 2020. For the center-right community, dominated by *Clarín*, *La Nación* and *Infobae*, we have $SB =-0.04 \pm 0.05$ in 2019 and $SB = -0.13 \pm 0.05$ in 2020. Sentiment bias values are shown in Table 4

**Table 4:** Sentiment bias values of the sum of all Center-left and Center-right communities of news networks in both years. Bold asterisks denote that sentiment bias values are significantly different from zero with $p_{\mathrm{value}} <0.01$

Sentiment bias		
Communities	Year	
	2019	2020
Center-left group	0.17 ± 0.05^∗^	0.02 ± 0.05
Center-right group	−0.04 ± 0.05	−0.13 ± 0.05^∗^

The first observation is that the difference between the SB of the two main groups persists in time. We quantified the statistical significance of these differences in two ways. First, we computed the Wilcoxon rank-sum test [51]. For 2019 and for 2020 the hypothesis was rejected at 1% significance level ($p_{\mathrm{value}}<0.01$). Second, we generated mean value distributions by bootstrapping with repositioning the sentiment bias sets. The null hypothesis was that both distributions had the same mean value. As with the Wilcoxon test, we obtained $p_{\mathrm{value}}<0.01$ for both years.

We also tested if the obtained values were significantly different from zero. Here, we can observe a clear difference between both years: on one hand, in 2019 $SB>0$ ($p<0.01$) in the community dominated by *Página 12* and *El Destape* (bias towards *Kirchner-Fernandez* or against *Macri*), and no significant bias in the community dominated by *Clarín*, *La Nación* and *Infobae*. It is important to bear in mind that 2019 was the year when Alberto Fernandez and Cristina Kirchner won the elections. On the other hand, in 2020, $SB>0$ ($p<0.01$) in the community dominated by *Clarín*, *La Nación* and *Infobae* (bias towards Macri or against Kirchner-Fernandez) and no significant bias in the community dominated by *Página 12* and *El Destape*. Thus, these results suggest a displacement in the support to the center-left coalition in 2019 towards the center-right in 2020, when Fernandez and Kirchner were the incumbent president and vice-president during the SARS-COV-2 pandemic year 2020.

### The user projection

In this projection, nodes represent users and edge weights are proportional to the number of news co-shared by these users. In the previous section we found that the identity of the emergent structures was mainly driven by the distribution of media outlets shared by each community. Here, we complement the topological analysis of networks with a description of each user in terms of its media consumer profile, which is given by the corrected media distribution defined in Eq. (5).

**Figure 5:**
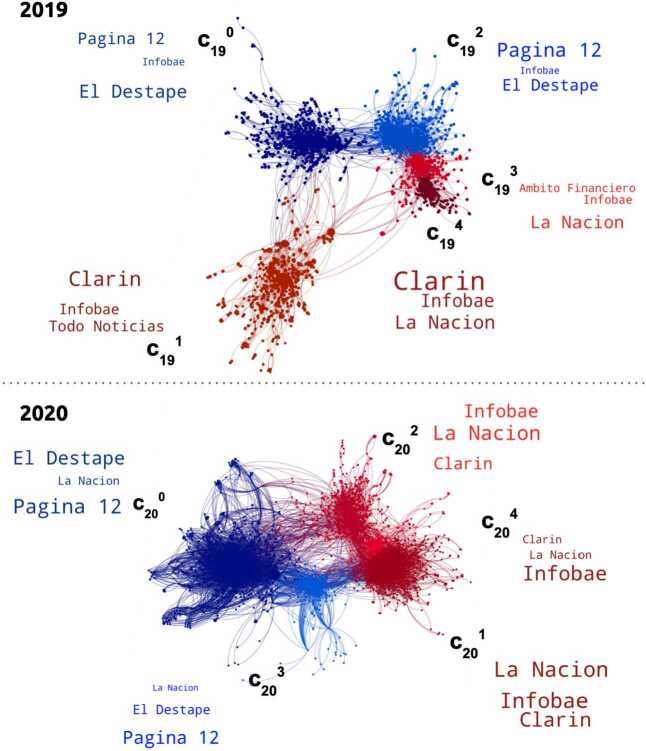
2019 and 2020 user networks visualization. Nodes were colored by communities membership. Word clouds display the averaged corrected media distribution of each community

We performed community detection in the datasets corresponding to both years. In Fig. 5 we can see highly structured user networks for the years 2019 and 2020. Nodes were colored according to their community membership, keeping red tones for center-right outlets and blue tones for center-left ones). The labels correspond to the average communities media vectors, according to Eq. (5) ($< m^{i}_{c}>$). In Table 5 we describe the relative sizes and the main media outlet vectors for the five largest communities. We can observe two different groups of communities: one with users who, in average, read news from *Página 12* and *El Destape*, and another with users who read news from *Clarín*, *La Nación* and *Infobae*.

**Table 5:** Main communities features for 2019 and 2020 users networks

Nodes (%)	Main Consumed Media Outlets
2019	
15.86	El Destape, Pagina 12
12.61	Clarin, Todo Noticias, Infobae, La Nacion
11.09	Pagina 12, El Destape
5.58	La Nacion, Infobae, Ambito Financiero, Pagina 12
5.31	Clarin, La Nacion, Infobae
2020	
16	Pagina 12, El Destape
11.17	La Nacion, Infobae, Clarin
8.11	La Nacion, Infobae, Clarin
7.35	Pagina 12, El Destape
7.12	Infobae

This visualization suggests that users form well-defined groups, as previously reported, and that they are connected largely due to the media they share in Twitter. Next, we investigated the appearance of homogeneous and well-differentiated communities, in the sense of the media consumer profiles.

**Figure 6:**
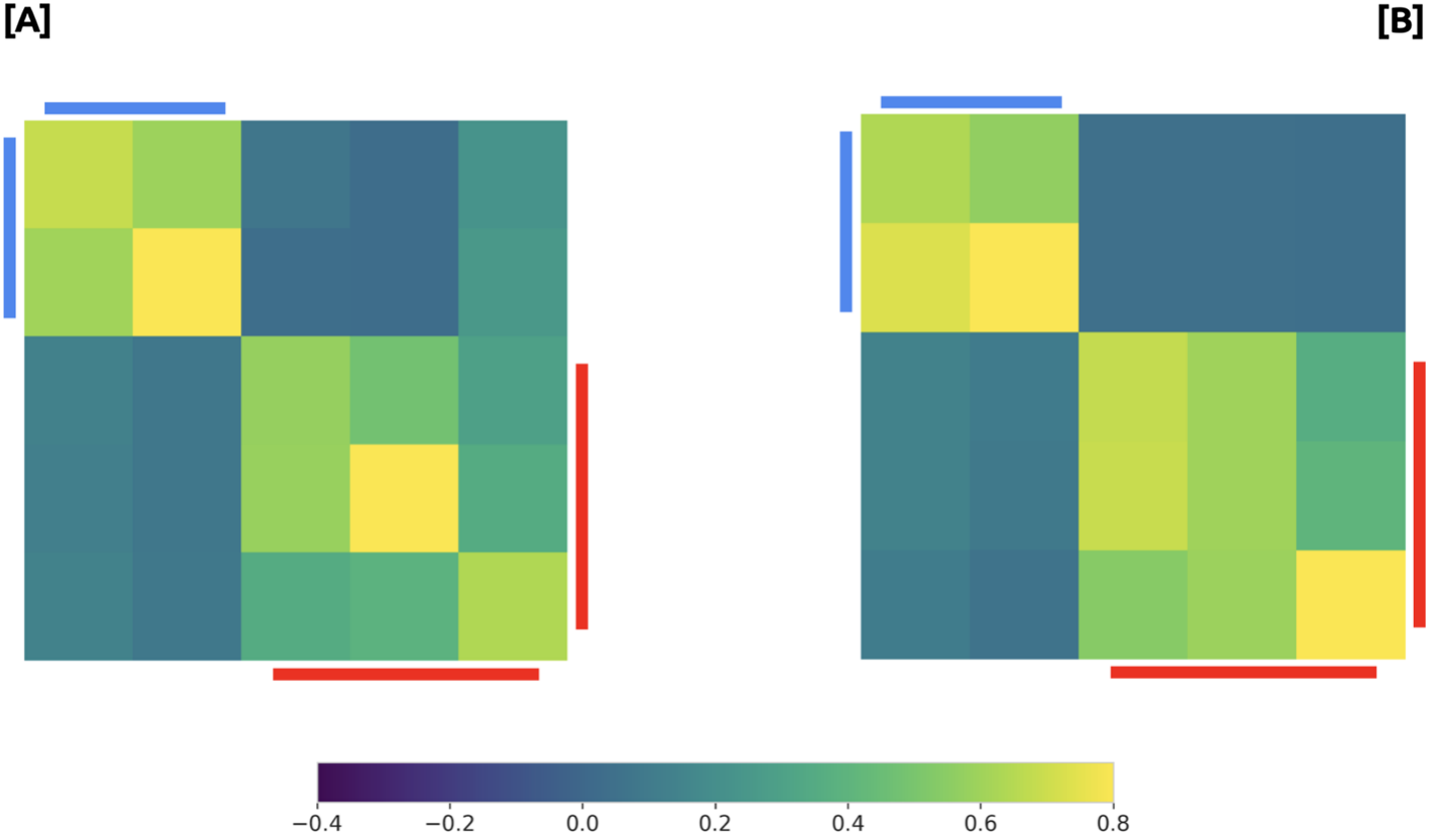
Similarities between users and average communities in media-consumed vectors. [**A**] and [**B**] accounts for 2019 and 2020 data sets, respectively. The i,j-th element of each figure corresponds to compute de median of the cosine similarities distribution between the average media-consumed vector of the i-th community and all the users media-consumed vectors belongin to the j-th community

In Fig. 6 we show the median of the cosine similarity distributions (computed between the average media vectors of users and communities). Here, the comparison with their own communities are sketched in the diagonal, while the comparison with other communities can be found outside the main diagonal. These results show the consistency of these communities in terms of media consumer profiles. We observed that the largest five communities could also be separated in two well differentiated groups: one dominated by *Clarin - La Nacion - Infobae*, and the other by *Pagina 12* and *El Destape*.

**Figure 7:**
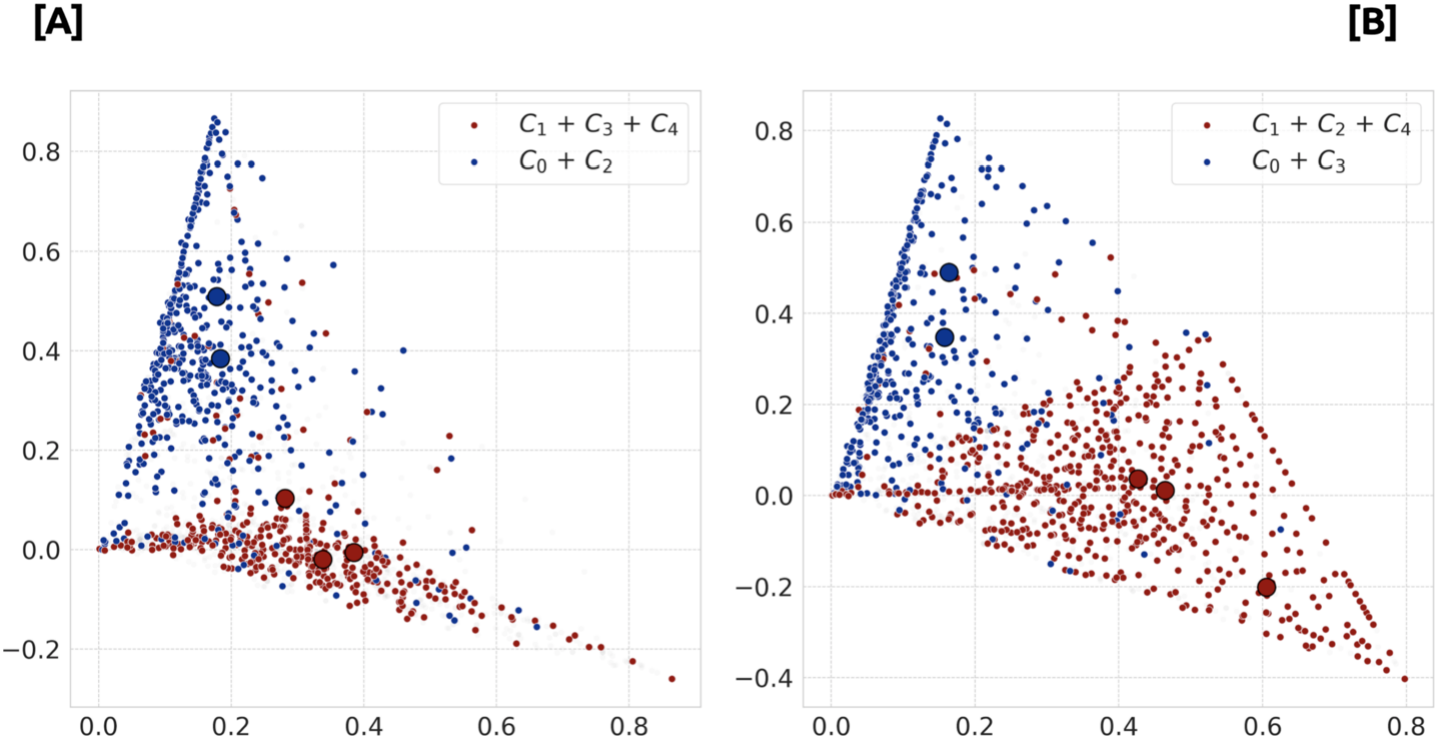
2020 and 2019 users corrected media vector mapping, after SVD transformation. Users belonging to communities identified previously as a block are coloured with the same color

In Fig. 7, average vectors for users and communities are embedded onto the first two dimensions of the Singular Value Decomposition (SVD) representation and colored according to their corresponding communities. Here, we observe that the differences between the center-left and the center-right groups are not only at the level of averages, but also between populations.

These results show that Argentinian media consumption in Twitter is polarized between two groups of users that reflect the political confrontation in national elections between a center-left and a center-right political coalition.

#### Diversity in the news consumption and their role in the communities

Previous results show that the users tend to cluster in communities according to the media outlets they read or share. We analyzed here if the lack of diversity in media consumption (as defined in Eq. (6)) plays an important role in the formation of these communities.

**Figure 8:**
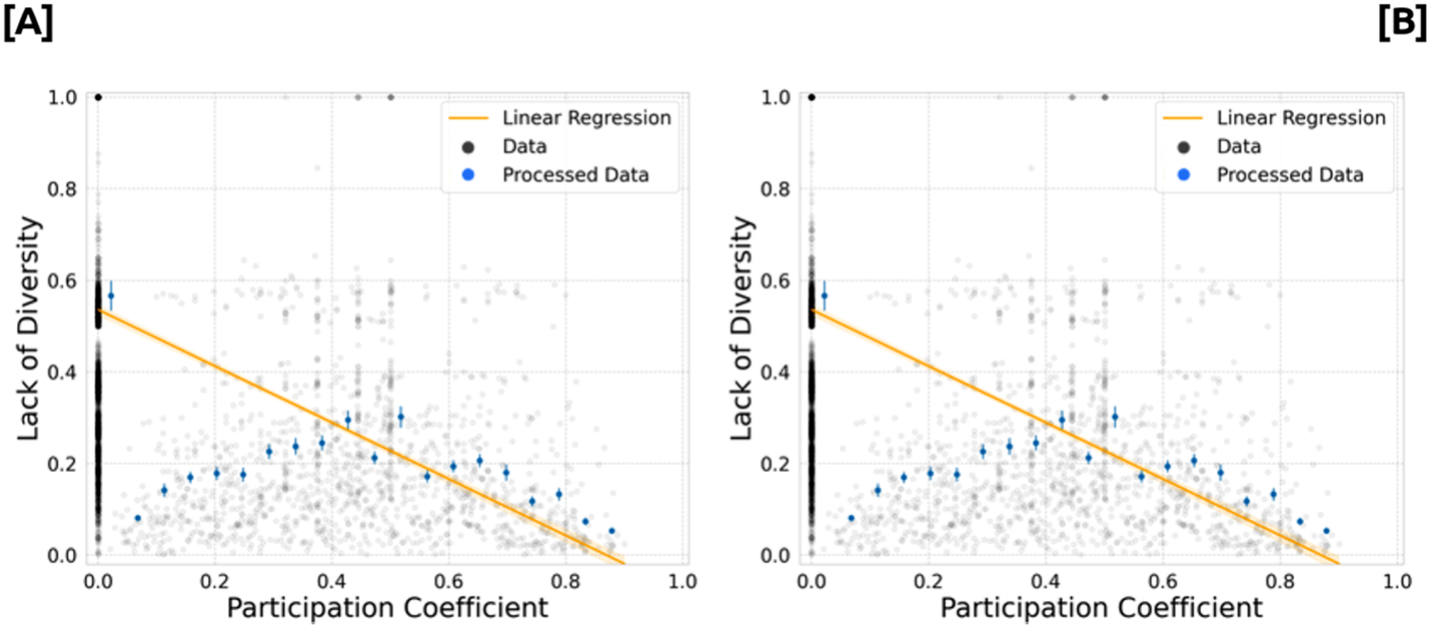
Lack of diversity vs participation coefficient. [**A**] and [**B**] display 2019 and 2020 data, respectively

In Fig. 8, we compare the lack of diversity in media consumption with the role of the users in the networks. In particular, we chose the participation coefficient defined because its low values indicate a strong membership to a given module.

Figure 8 shows that there is a negative correlation between the participation coefficient and the lack of diversity of the users in both data sets. The values of the linear correlations for 2019 and 2020 are −0.47 and −0.45, respectively ($p_{val} < 0.01$ compared to a null model where the *LD* values were randomly shuffled). However, it is clear that the negative correlation is determined by those users who have participation coefficient $pc = 0$, while users with low but non-zero *pc* present a different relationship with their *LD*. These results show that users that play a central role in each community have a noticeable lack of diversity in the news they consume. On the other hand, users that are in the border between communities tend to share news from different outlets.

## Discussion

The role of social media in the formation of groups of like-minded users that share a given narrative (known as echo chambers) has been extensively studied and is of considerable interest, given its profound impact in the formation of public opinion.

The emergence of echo chambers has been observed in networks defined by preferential message propagation (*retweet networks*, in the case of Twitter) [11, 12], in networks of followers [13], as well as in discussions around a single topic. They reflect the clustering of individuals based on different measures of similarity. For instance, in [13] groups were identified by the leaning of the media they tweeted; in [15, 18], according to the ratio of positive vs. negative votes on the discussed topic in Facebook; in [14] they were defined by common hashtags, and in [16] by following similar groups of influencers.

Our results are also relevant to the analysis of ideological polarization processes, where groups are formed by ideological affinity [24], which consists in sharing opinions on an ample variety issues [22].

Given the importance of the media in the processes of opinion formation [1, 2] and considering how the affinity ties between users limit and constraint the circulation of information [8], some relevant questions are: what is the role of the media in the emergence of these groups? Which is the role of the underlying political polarization in this process?

We studied whether news sharing of Argentinian newspapers in social media produced the emergence of homogeneous groups in terms of media consumption habits. Our main contribution is the detection of echo chambers in bipartite user-news networks and their identification in terms of consumption patterns of media outlets associated with the underlying ideological patterns characteristic of Argentinean political life.

We selected two sets of Twitter users and focused on tweets with links to media outlets. With this data, we built a bipartite network of users and news and analyzed their projections in both layers, focusing on the analysis of emergent structures at a meso-scale level (communities) and the role of nodes in these structures. We compare the results between an electoral and non-electoral year and between a set of politically active users and a control group.

The main result is the emergence of two main groups of news articles identified by a unique distribution of media outlets in all analyzed datasets. On one side, news from *Página 12* and *El Destape* appear to be highly connected in one group (the center-lef one) and news from *Clarín*, *La Nación* and *Infobae* in the other (center-right). This division is consistent with the ideological leaning of some of these outlets, as was previously reported in [30–32]. The same distribution was found in the set of control users, indicating that these results do not depend if users show explicit political preferences. Figure S6 in Additional file 1 shows the similarities between these two groups across datasets.

We also verified if the ideological leaning of the media outlets paralleled the one observed in the two main political Argentinian coalitions which disputed the national elections in 2019. This was done by means of sentiment analysis applied to the news articles belonging to the two main groups. The obtained values of the Sentiment Bias Statistics (SB) show consistently a significant difference between these two groups in both years. An interesting results was a change from a sentiment bias towards the candidates of the center-left coalition (or against the candidates of the center-right one) in 2019 to zero in 2020 in the groups of news articles dominated by *Página 12* and *El Destape*. A similar displacement in the values of the sentiment values from zero to a bias towards the candidates of the center-right coalition (or against the candidates of the center-left one) appeared in the groups of news articles dominated by *Clarín*, *La Nación* and *Infobae*. A possible interpretation of these results is that in 2019, the candidates of the center-left coalition (Alberto Fernandez and Cristina Kirchner) won the national elections (and therefore a bias towards the winning candidates), meanwhile 2020 was a year dominated by the SARS-COV-2 pandemic, and certain measures (such as prolonged quarantines) were detrimental for the incumbent president.

As expected, the analysis of the user projection network produced similar results. The news-sharing behavior gave rise to the emergence of two defined groups of users which could be defined in terms of the distribution of news from the media they shared: one of them dominated by *Clarin - La Nacion - Infobae* and the other by *Pagina 12* and *El Destape*.

Our results contribute to the characterization of echo chambers in terms of vector-media consumption, and to the use of sentiment bias to infer the ideological leaning of media outlets. They also shed light on the process by which the political polarization in Argentina constrain the exposure to media content in social media. We should point out, however, that the sets of selected users, as well as the time span of their activity, clearly constraint the range of our conclusions. Another limitation is the use of the Louvain algorithm to detect communities. Even though we checked our results with other algorithms obtaining similar results, further analysis may include other alternatives, such as stochastic block models. Future work should expand these analysis to larger datasets (comprising longer periods of time) and to other countries. In particular, the definition of ideological leaning should be checked with different measures to test its robustness and consistency.

## Supplementary Information

Below is the link to the electronic supplementary material.

Supplementary information (PDF 4.7 MB)

## Data Availability

The datasets supporting the conclusions of this article are available in the GitHub repository, in https://github.com/LMDC-DF/twitter_mediaConsumption

## Footnotes

1 The sub-index *c* stands for *corrected*
