# Supplementary material for: News sharing on Twitter reveals emergent fragmentation of media agenda and persistent polarization
Source: EPJ Data Sci. 2022 Aug 19;11(1):48. doi: 10.1140/epjds/s13688-022-00360-8 (PMC9388975; doi:10.1140/epjds/s13688-022-00360-8)
Supplement: Supplementary file 1 — Supplementary information (PDF 4.7 MB) [file 13688_2022_360_MOESM1_ESM.pdf]

Supplementary Information for: News-sharing on Twitter reveals emergent fragmentation of media agenda and persistent polarization

Tomas Cicchini[1,3], Sofia M. del Pozo[1,2], Enzo Tagliazucchi[1,2], and Pablo Balenzuela *[1,2]

[1]Departamento de Física, Facultad de Ciencias Exactas y Naturales, Universidad de Buenos Aires. Av.Cantilo s/n, Pabellón I, Ciudad Universitaria, C1428EGA, Buenos Aires, Argentina.

[2]Instituto de Física de Buenos Aires (IFIBA), CONICET. Av.Cantilo s/n, Pabellón I, Ciudad Universitaria, C1428EGA, Buenos Aires, Argentina.

[3]Instituto del Cálculo (IC), UBA-CONICET. Intendente Güiraldes 2160, Ciudad Universitaria, Pabellón II, 2do. piso, C1428EGA, Buenos Aires, Argentina.

June 16, 2022

1 Keywords for the Twitter search of users

In order to select both set of users (political engaged and those who mention media outlets), we use the list of keywords listed in Table 1. Those users who tweeted using some of this words where selected.

2 Bipartite Degree for News and Users

The degree distributions of the bipartite networks for users and news are shown in Fig. S1. As it can be seen, the long tail distributions implies that there are users that share compulsively news, as well as there are news that are consumed multiple times. Therefore, an hyperbolic projection turns naturally, to mitigate the effects of this highly connected nodes.

*balen@df.uba.ar

User dataset	Keywords tweeted
Politically active users	Hashtag list: Elisacarrio, OfeFernandez_, PatoBullrich, macri, macrismo, mauriciomacri, pichetto, MiguelPichetto, JuntosPorElCambio, alferdez, CFKArgentina, CFK, kirchner, kirchnerismo, FrenteTodos, FrenteDeTodos, Lavagna, RLavagna, Urtubey, UrtubeyJM, ConsensoFederal, 2030ConsensoFederal, DelCaño, NicolasdelCano, DelPla, RominaDelPla, FitUnidad, FdeIzquierda, Fte_Izquierda, Castañeira, ManuelaC22, Mulhall, NuevoMas, Espert, jlespert, FrenteDespertar, Centurion, juanjomalvinas, Hotton, CynthiaHotton, Biondini, Venturino, FrentePatriota , RomeroFeris, PartidoAutonomistaNacional, Vidal, mariuvidal, Kicillof, Kicillofok, Bucca, BuccaBali, chipicastillo, Larreta, horaciorlarreta, Lammens, MatiasLammens, Tombolini, matiastombolini, Solano, Solanopo, Lousteau, GugaLusto , Recalde, marianorecalde , RAMIROMARRA, Maxiferraro , fernandosolanas , MarcoLavagna , myriambregman , cristianritondo, Massa, SergioMassa , GracielaCamano, nestorpitrola
Users control group	La Nación, Clarín, Infobae, Página 12, El Destape, La Izquierda Diario, Ámbito Financiero, Radio Mitre, Minuto Neuquén, Rosario 3, El Cronista, El Chubut

Table 1: *Keyword lists used to select users.*

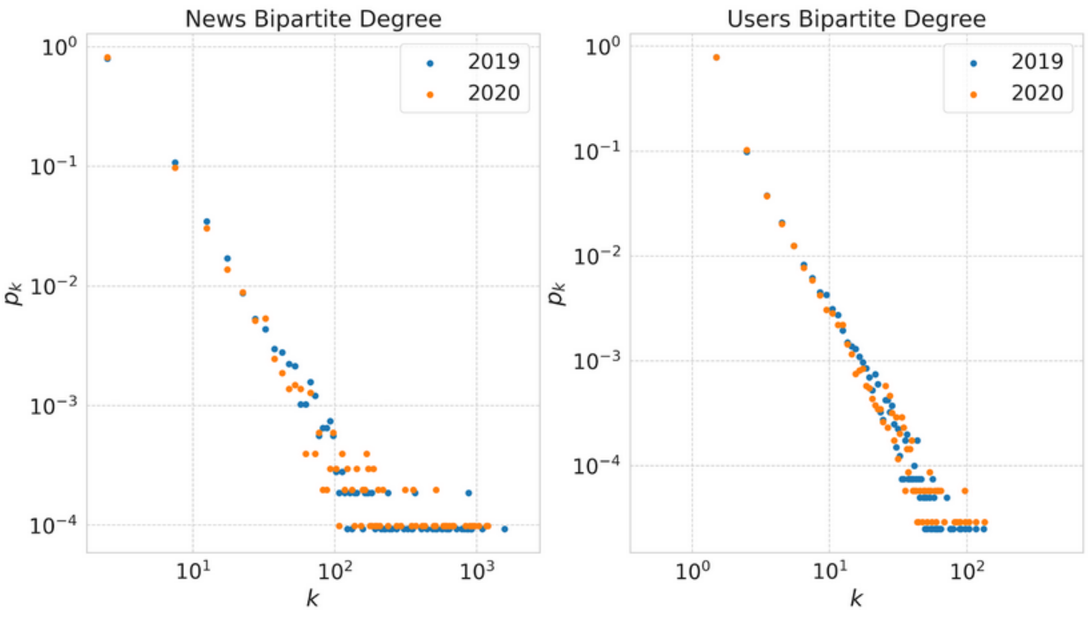

Figure S1: *Degree of news and users in the bipartite networks. On the one hand, the user degree refers to the number of news shared by the user; on the other hand, news degree stands for the number of users that shared a new.*

3 Summary of Networks Topology

In Table 2, we provide a summary of different topological properties of all analyzed networks. Although the amount of users selected in both datasets was the same, after discarding tweets that do not contain media outlet URLs, differences between them can be seen. Networks from politically active dataset are larger and less dense than those from control group. On one hand, control group news networks present less edges (E) and mean degree ($< k >$) than the politically active networks. On the other hand, control group users networks have more edges and $< k >$ than politically active ones.

	**Users Networks**				**News Networks**			
	**Politically active**		**Control Group**		**Politically active**		**Control Group**	
**Year**	**2020**	**2019**	**2020**	**2019**	**2020**	**2019**	**2020**	**2019**
**N**	4323	3625	3021	1844	10975	12221	6597	4734
**E**	15621	10154	18603	5718	83611	111422	22140	12283
**<k>**	7.23	5.60	12.32	6.20	15.24	18.23	6.71	5.19
**<s>**	3.38	3.99	3.10	2.85	1.36	1.34	1.35	1.17
**Average Clustering**	0.30	0.24	0.31	0.25	0.51	0.52	0.40	0.35
**Density**	0.0017	0.0015	0.0041	0.0034	0.0014	0.0015	0.0010	0.0011

Table 2: *Summary of the topological properties of the networks from both data sets. PASO and Control Group refer to the political engaged users data set and the users that mentioned media outlets data set, respectively.*

4 Normalized Mutual Information between Partitions

In order to compare communities obtained from Louvain community detection algorithm, normalized mutual information was performed between the 20 main communities from partitions detected by three different methods: Louvain [1], Infomap [2] and Label Propagation [3]. The results of this analysis are shown in Table 3. As it can be seen, the detected communities are similar in terms of normalized mutual information in all cases.

<table>
<tr><th colspan="2" rowspan="2">News Networks</th><th colspan="2">2019</th><th colspan="3">2020</th></tr>
<tr><th>Louvain</th><th>Infomap</th><th>Infomap</th><th>Louvain</th><th></th></tr>
<tr><td rowspan="2">2019</td><td>Label propagation</td><td>0.83</td><td>0.78</td><td>0.91</td><td>0.93</td><td rowspan="2">2020</td></tr>
<tr><td>Infomap</td><td>0.92</td><td>-</td><td>-</td><td>0.87</td></tr>
<tr><th colspan="2" rowspan="2">Users Networks</th><th colspan="2">2019</th><th colspan="2">2020</th><th></th></tr>
<tr><th>Louvain</th><th>Infomap</th><th>Infomap</th><th>Louvain</th><th></th></tr>
<tr><td rowspan="2">2019</td><td>Label propagation</td><td>0.70</td><td>0.76</td><td>0.82</td><td>0.89</td><td rowspan="2">2020</td></tr>
<tr><td>Infomap</td><td>0.89</td><td>-</td><td>-</td><td>0.82</td></tr>
</table>

Table 3: *Normalized mutual information score between the partitions of the 20 main communities obtained from applying Louvain, Infomap and Label Propagation algorithms on both, users and news, networks.*

5 Topic Description

In the main work, a topic decomposition of the news content in the set of politically active users was performed. In this section, word clouds for each topic of the main two communities of news for both years are shown. It should be noticed that in both 2019 communities, a topic related with the **National Election** emerge. Among the most frequent words (in terms of tf-idf) appears the names of the candidates of the two main political coalitions on the electoral dispute. This co-appearance aims us to compute the sentiment bias, as mentioned on the main work.

6 Analysis of the Control Data Set

As mentioned in the main work, a control data set was analyzed in order to asses the robustness of our original results. Analysis for both, users and news networks, can be found on the next subsections.

6.1 The news projection

Firstly, we present the results of the analysis of the news projection of the control group data set. In Fig. S4, a visualization of both, 2019 and 2020, networks are shown. As before, two main communities emerge, were each one is dominated by a given group of media outlets: on the one hand, *Pagina 12* and *El Destape*; and on the other hand, *Clarin*, *Infobae* and *La Nación*.

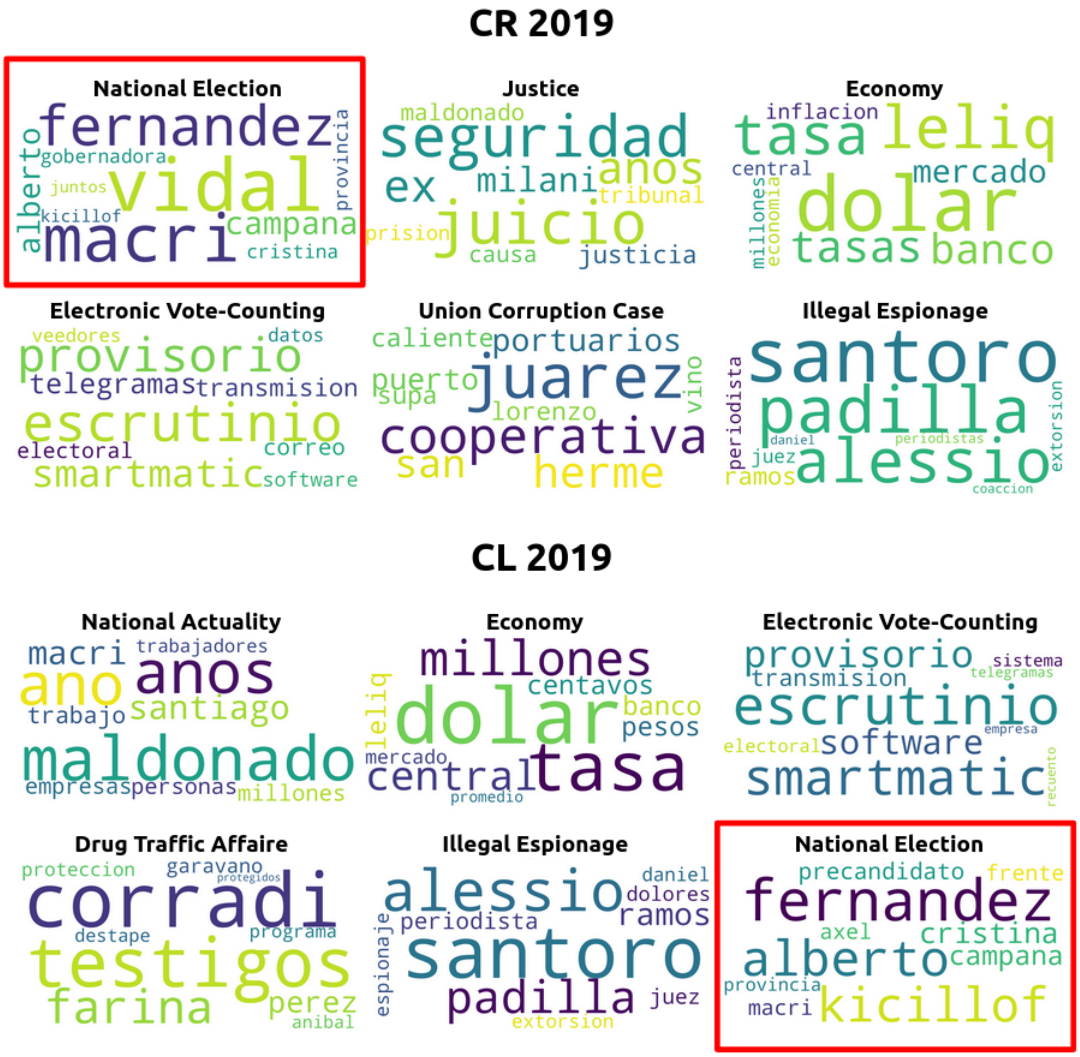

Figure S2: *Topic description on the term space, mapped on word clouds, where the size of each word is proportional to the weight of the word on each topic. Center right and center left main communities of 2019 news network are shown. Word clouds boxed in red highlight the National Election topics of both communities, were candidate names appear.*

Motivated by the previous network visualizations, we compute the cosine similarity between the media outlet distribution of the main communities. This similarity was computed between communities of the same year and also between communities of different years. As it is shown in Fig. S5, it is possible to identify two groups of communities: the Center-Right ones and the Center-Left ones.

Then, for the main two communities of each year, a media outlet distribution and a topic decomposition of the news content analysis were performed. Results are presented in Fig. S6. It is notorious that both communities are dominated by different set of media outlets, as highlighted before.

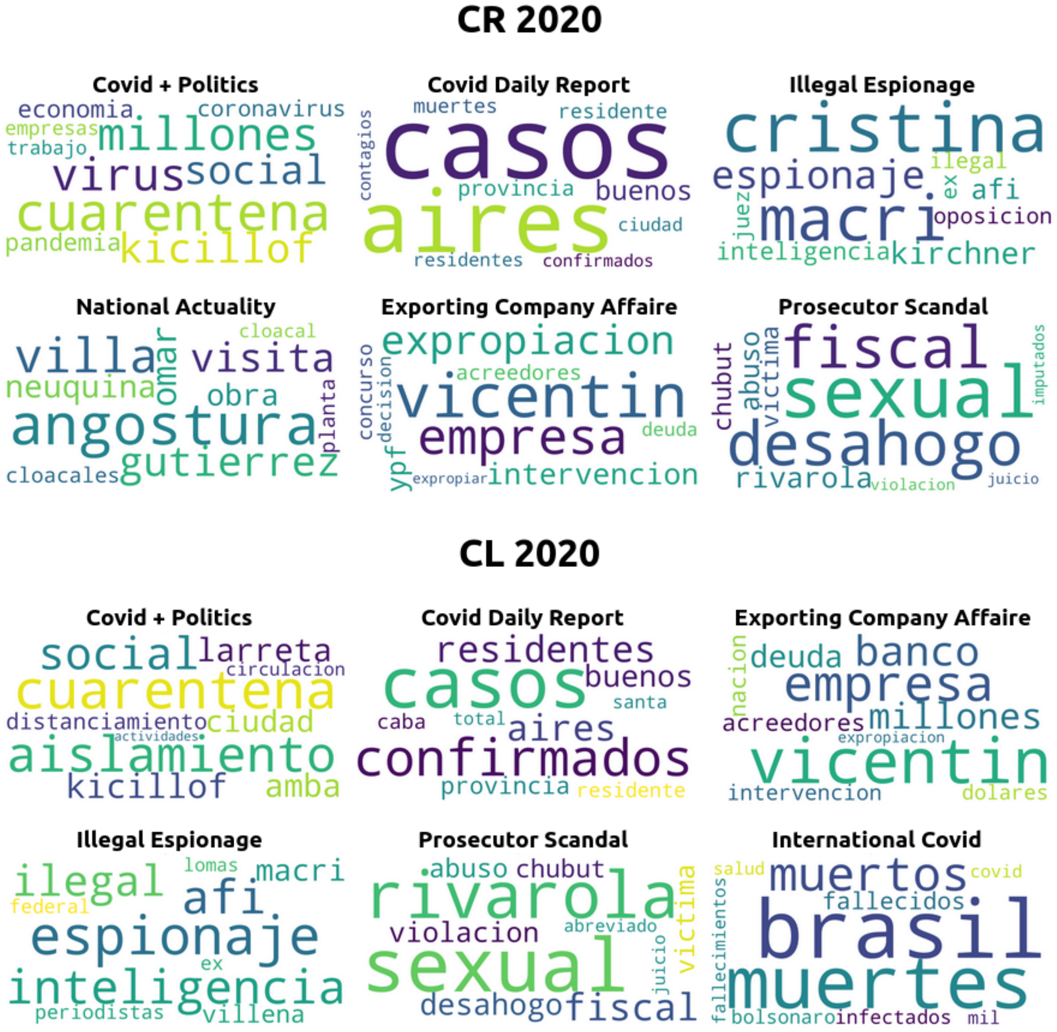

Figure S3: *Topic description on the term space, mapped on word clouds, where te size of each word is proportional to the weight of the word on each topic. Center right and center left main communities of 2020 news network are shown.*

Finally, we decide to compare the media outlet distributions between the main five communities of the control group news networks and the main five communities of the politically active news networks. The computed cosine similarities are shown in Fig. S7

We cab appreciate here the high degree of similarity in center-left and center-right groups between both datasets.

6.2 The users projection

Now, we present the results of the analysis of the users projection of the control group data set. In Fig. S8, a visualization of 2019 and 2020 networks are shown, along with the corresponding word clouds that represent the average media-consumed vector of the main communities.

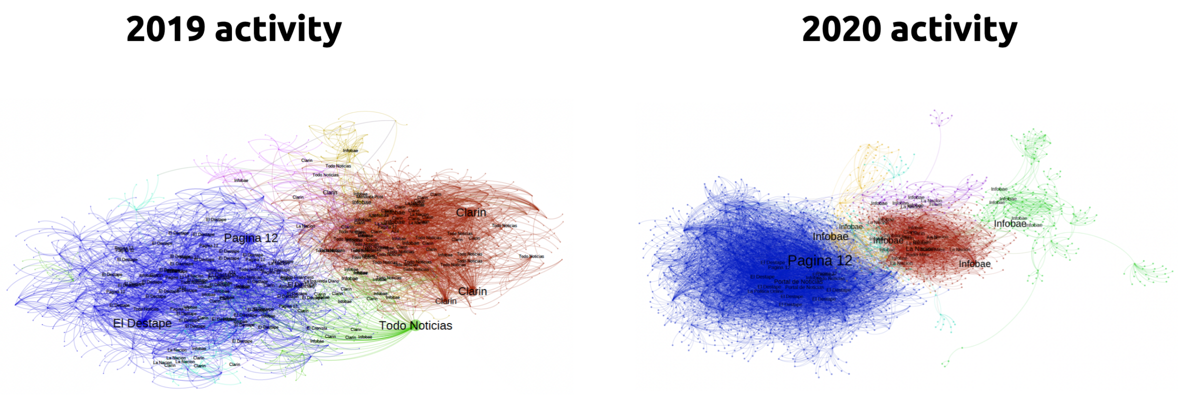

Figure S4: *2019 and 2020 news networks visualization. Nodes were colored by community membership and labeled by media outlet proportionally to their within-module degree.*

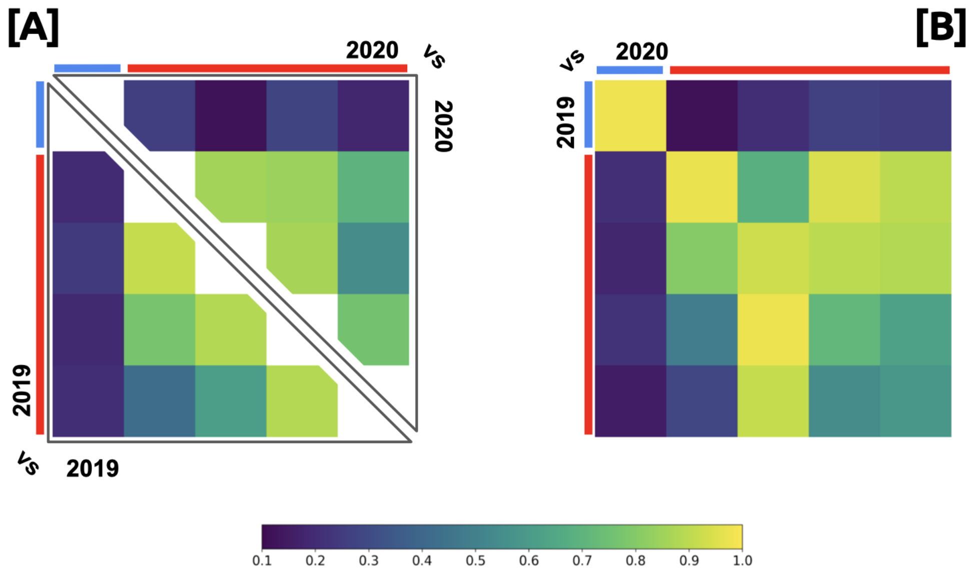

Figure S5: *Consistency of news network. Panel [A] accounts for similarities among communities of same years and Panel [B] compare 2019 against 2020.*

Communities where colored by users news consumption. Those communities in which users tweeted mainly with links to center-left media outlets were colored in red and those with links to center-right outlets in blue.

In Fig. S9, we compute the similarities between the average media-consumed vector of the main communities and the media-consumed vector of the users, for both years. As seen in the main analysis, a group of Center-Right and a group of Center-Left communities emerge.

And finally, the 2020 and 2019 users corrected media vector mapping is shown in Fig. S10. Those users previously detected in the same community are colored with the same color, red for center-right community and blue for center- community. It can be seen a similarly communities structure than politically active users network has. Also there is a clear difference in media

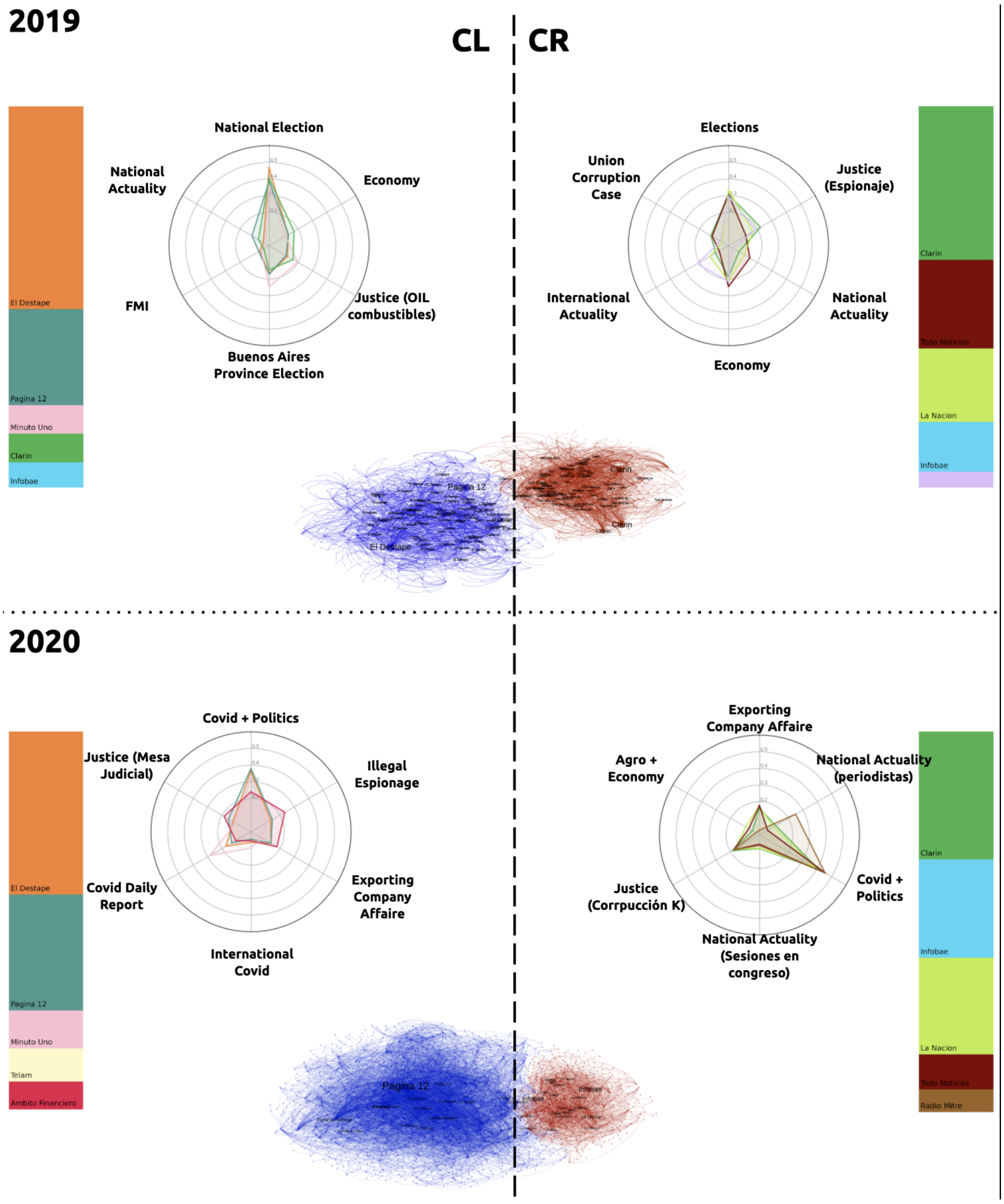

Figure S6: *Media outlets distributions and topic decomposition for the 2019 ans 2020 two main communities. The stacked bars represents the media outlet distribution, while the radar plot display the media agenda. The different colored lines in radar plot indicate the agendas of each outlet with the same color as in the stacked bar.*

consumption depending on the user communities

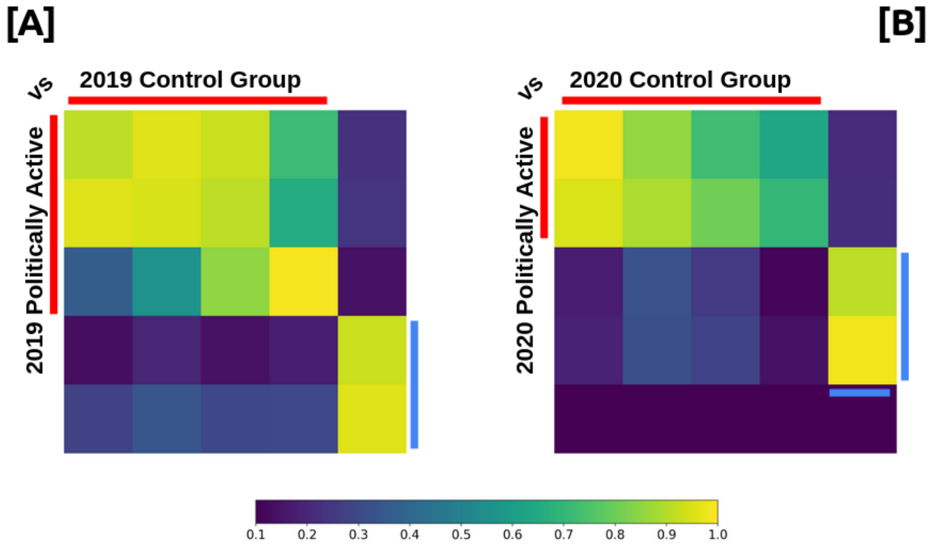

Figure S7: *Cosine similarities between the main five communities of the politically active and control group news networks. 2019 networks are compared on panel [A], while 2020 similarities are shown on panel [B].*

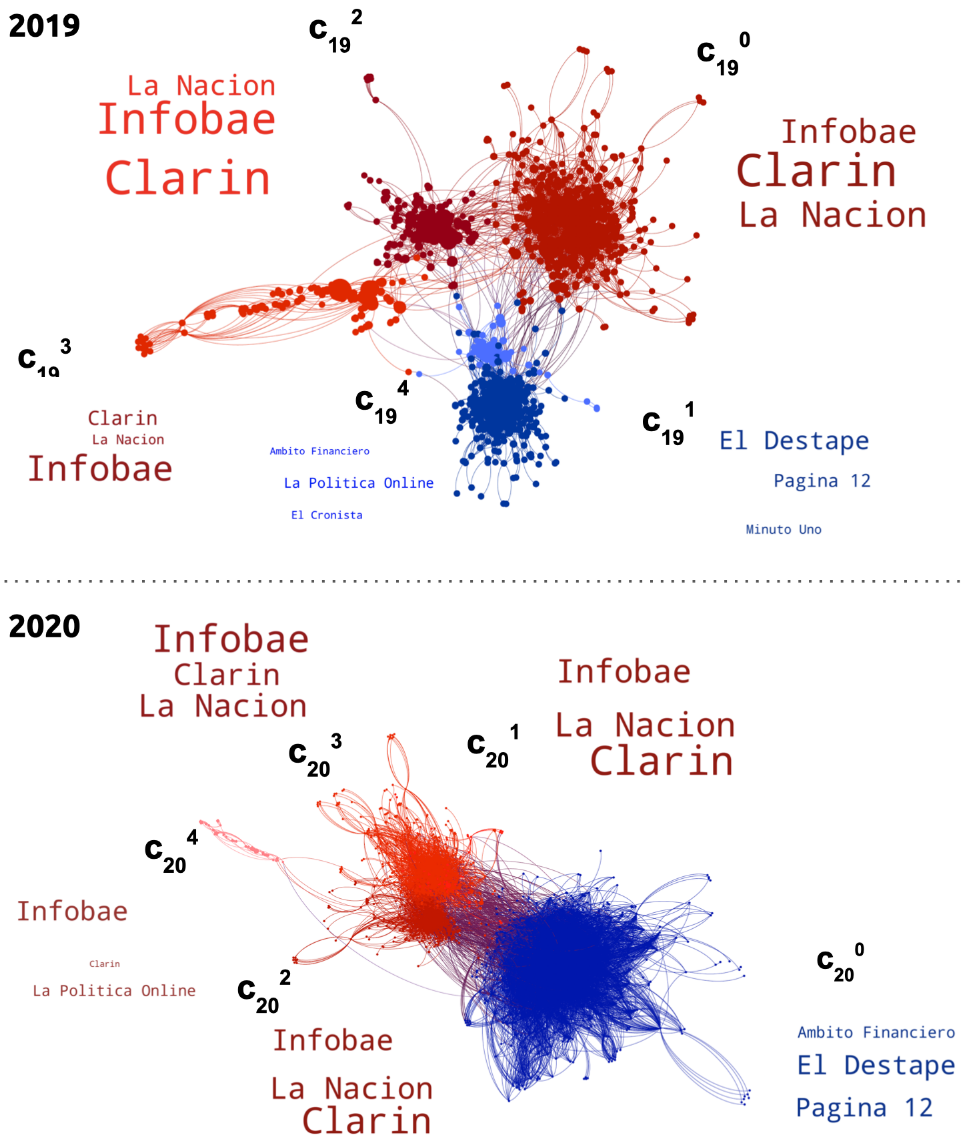

Figure S8: *2019 and 2020 user networks visualization. Nodes were colored by communities membership. Word clouds display the averaged corrected media distribution of each community.*

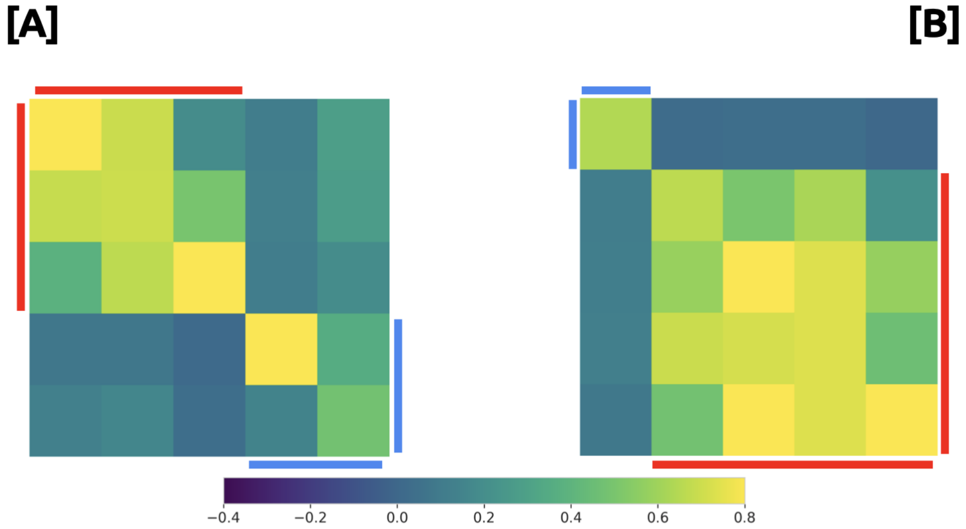

Figure S9: *Similarities between users and average communities in media-consumed vectors. [A] and [B] accounts for 2019 and 2020 data sets, respectively. The i,j-th element of each figure corresponds to compute de median of the cosine similarities distribution between the average media-consumed vector of the i-th community and all the users media-consumed vectors belonging to the j-th community.*

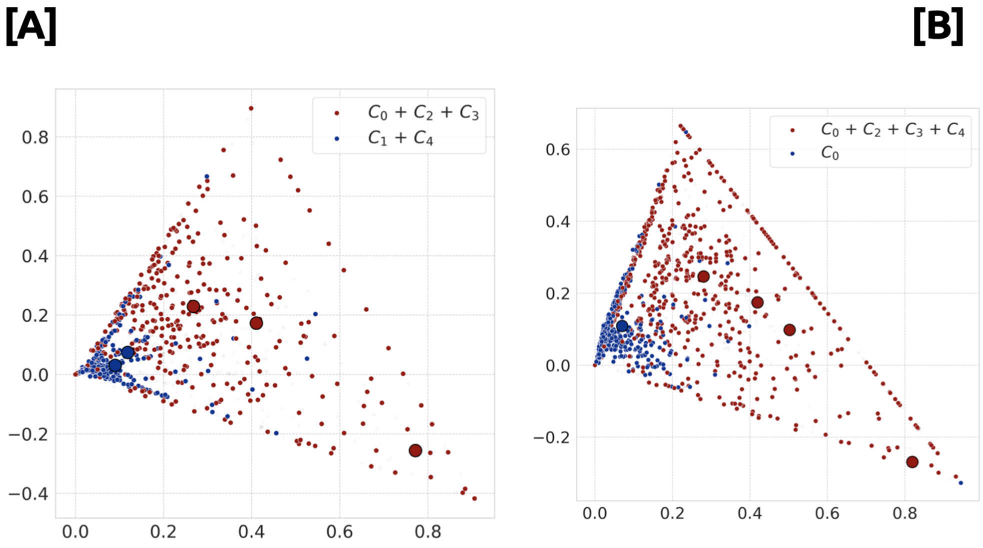

Figure S10: *2020 and 2019 users corrected media vector mapping, after SVD transformation. Users belonging to communities identified previously as a block are coloured with the same color.*

References

[1] Louvain community detection algorithm: https://github.com/taynaud/python-louvain

[2] Infomap community detection algorithm: https://igraph.org/python/doc/api/igraph.Graph.html#community_infomap

[3] Label propagation algorithm: https://igraph.org/python/doc/api/igraph.Graph.html#community_label_propagation
